# Supplementary material for: Oocyte mitophagy is critical for extended reproductive longevity
Source: PLoS Genet. 2022 Sep 20;18(9):e1010400. doi: 10.1371/journal.pgen.1010400 (PMC9524673; doi:10.1371/journal.pgen.1010400)
Supplement: S1 Fig — (A) Day 1 adult mitochondrial morphology images in the -1 oocytes of N2 (left 2 panels) and daf-2(e1370) (right 2 panels). (B) Day 7 adult mitochondrial morphology images in the -1 oocytes of N2 (left 2 panels) and daf-2(e1370) (right 2 panels). In A and B images were taken on the Nikon eclipse Ti at 60x magnification. (C) Day 7 adult mitochondrial morphology images in the muscle of N2 (left 2 panels) and daf-2(e1370) (right 2 panels). In C images were taken on the scanning confocal Nikon A1 at 60x. All images are of mitochondria stained with ATP5α to mark membranes. (PDF) [file pgen.1010400.s001.pdf]

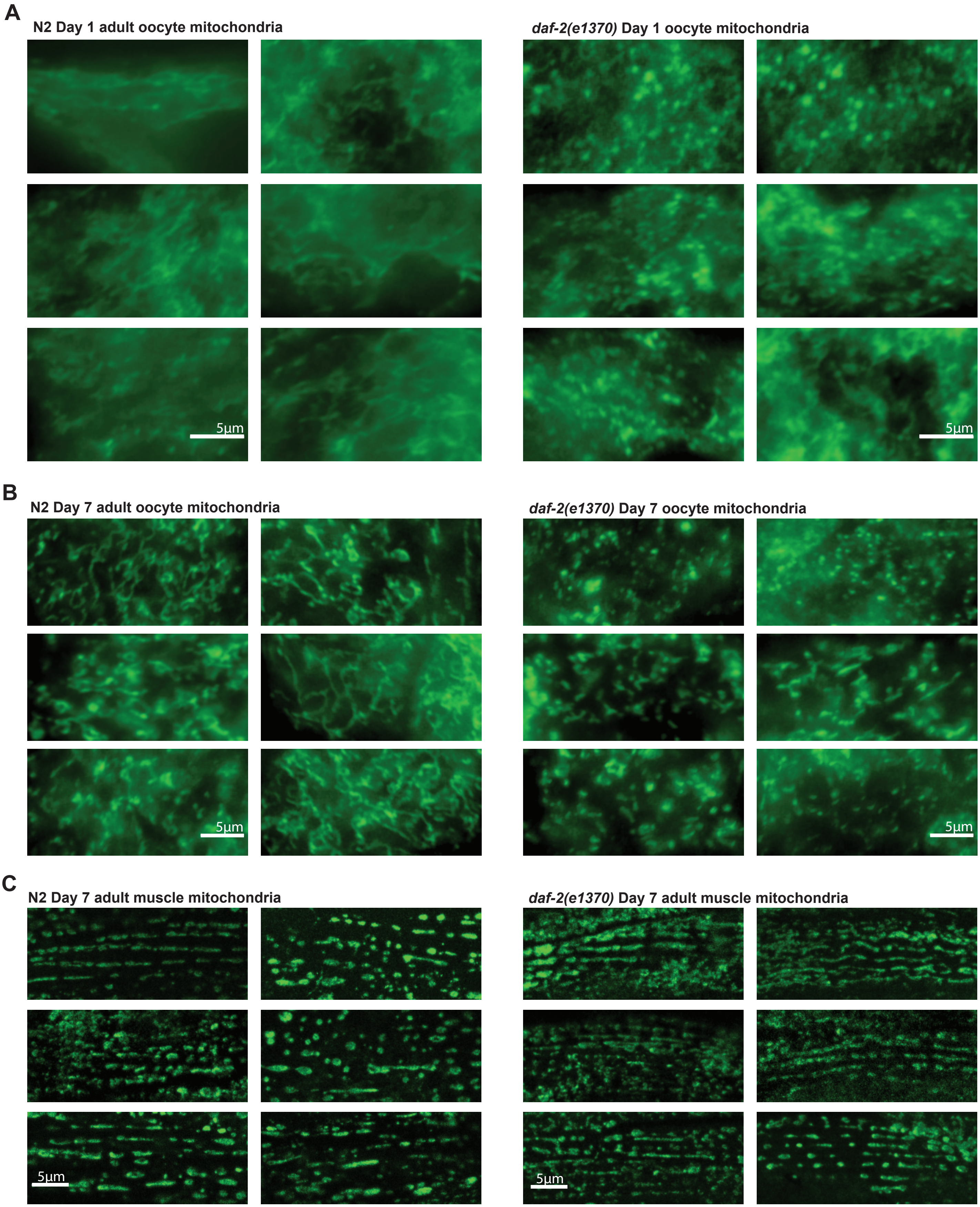

Supplemental Figure 1
