# Supplementary figures and images for: Oocyte mitophagy is critical for extended reproductive longevity
Source: PLoS Genet. 2022 Sep 20;18(9):e1010400. doi: 10.1371/journal.pgen.1010400 (PMC9524673; doi:10.1371/journal.pgen.1010400)

**A**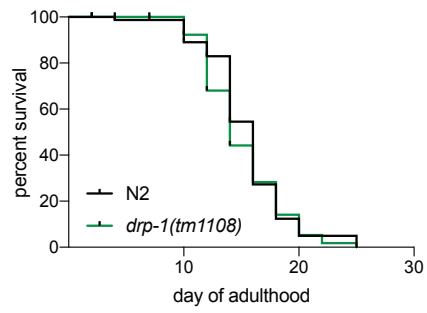**B**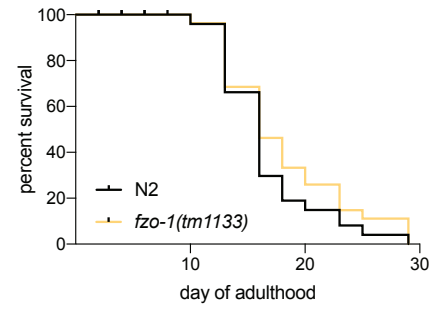**C**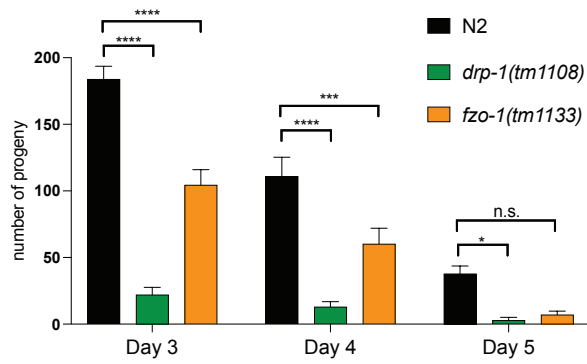**Supplemental Figure 2**

Supplement: S2 Fig — (A) Lifespans are not affected by loss of drp-1(tm1108) (n = 81, N2 n = 81) (B) fzo-1(tm1133) (n = 80, N2 n = 83). (C) Progeny count of mated N2 (n = 12), drp-1 (n = 13) and fzo-1 (n = 12) after mating (L4-Day 1) and after mating confirmation (Day 1-Day 2). Day 3, 4 and 5 of adulthood were scored. 2-way ANOVA. (PDF) [file pgen.1010400.s002.pdf]

**A**

"young" Day 5

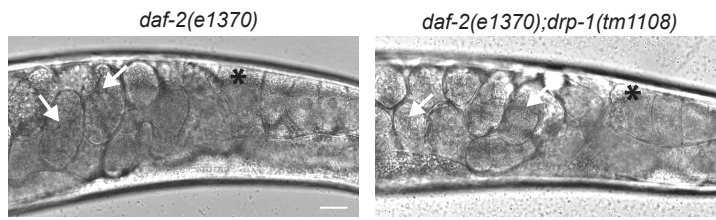**B**

"aged" Day 8

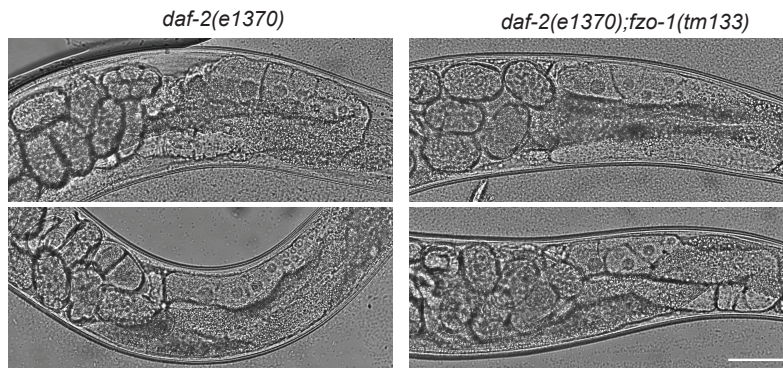**C**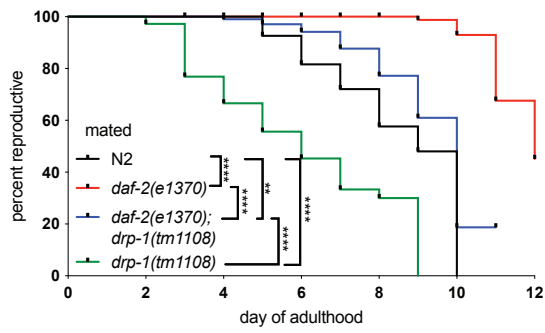**D**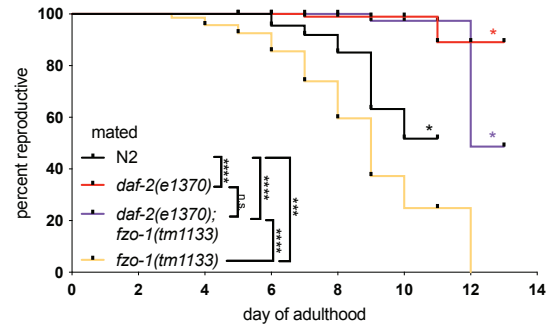

Supplement: S3 Fig — (A) Representative images of young daf-2;drp-1 and daf-2.Scale bar 20μm. (B) Representative images of aged daf-2(e1370) left and daf-2(e1370);fzo-1(tm1133) germlines. Scale bar 50μm. (C) Reproductive spans of daf-2 vs. daf-2;drp-1 (Fig 3D) including N2 and drp-1 genotypes. (D) Reproductive spans of daf-2 vs. daf-2;fzo-1 (Fig 3E) including N2 and fzo-1 genotypes. (PDF) [file pgen.1010400.s003.pdf]

**A***Ppie-1*TOMM-20::Rosella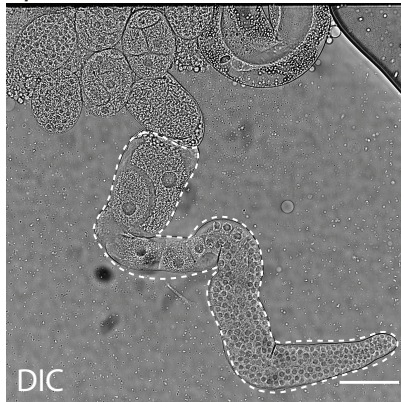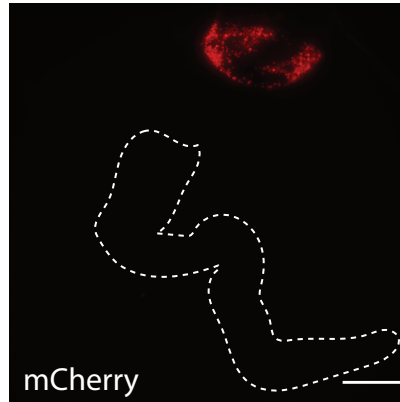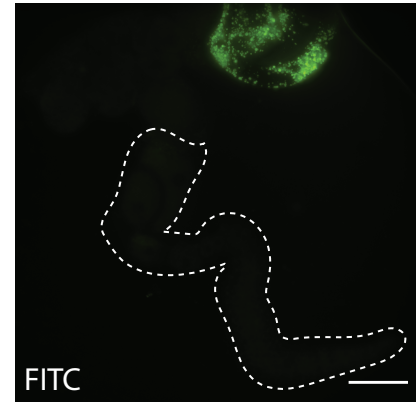

N2, wbmls60[pie-1p::TOMM-20::Rosella::unc-54 3'UTR, III:7007600]

**B**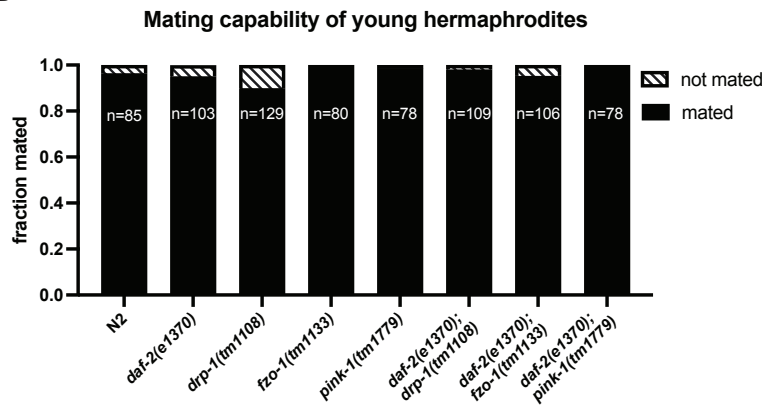**C**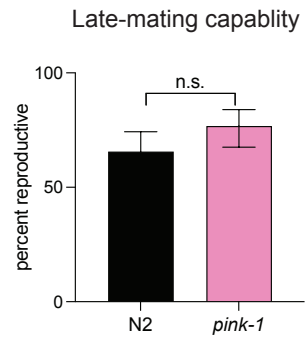**D**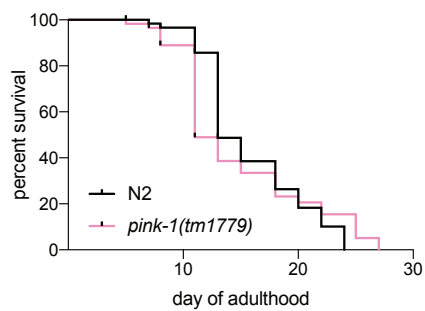

**Supplemental Figure 4**

Supplement: S4 Fig — (A) mtRosella is silenced in the germline. Image of the dissected germline of the WMB1119 strain with Ppie-1TOMM-20::Rosella insert. Image was captured on the Nikon eclipse Ti at 60x magnification. The mCherry and FITC channels are superimposed Z-stacks taken at 0.7μm steps. (B) Mating capability of all genotypes used in this study when young hermaphrodites are mated with fog-2 males. (C) Late-mating capability in pink-1 vs. N2. Day 7 adults are mated with young males. (D) Lifespan of pink-1(tm1779) (n = 62) is unchanged compared to N2 (n = 69). (PDF) [file pgen.1010400.s004.pdf]

**A**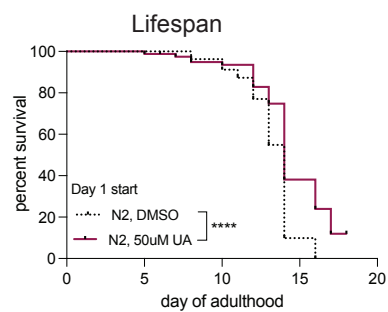**B**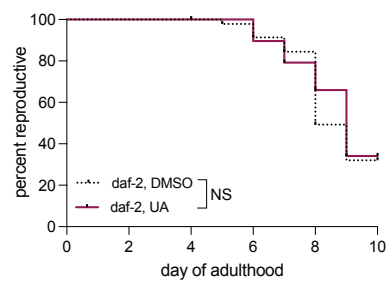

Supplement: S5 Fig — (A) Lifespan of N2 (unmated) hermaphrodites is extended with Urolithin A treatment (n = 100) vs. 0.3% DMSO (n = 100). Worms were cultured on NGM plates seeded with OP50 during development. Treatment started with or without Urolithin A on Day 1 and loaded in the CeLab chip. (B) daf-2 reproductive span does not improve with Urolithin A treatment. DMSO control (N = 50) vs. UA treated (n = 50). (PDF) [file pgen.1010400.s005.pdf]
