# Supplementary material for: Oocyte mitophagy is critical for extended reproductive longevity
Source: PLoS Genet. 2022 Sep 20;18(9):e1010400. doi: 10.1371/journal.pgen.1010400 (PMC9524673; doi:10.1371/journal.pgen.1010400)
Supplement: S1 Table — (A) Reproductive span, (B) Late-mating, and (C) Lifespan statistical information. Statistics were performed on Prism. Mean reproductive/alive was performed on OASIS. (DOCX) [file pgen.1010400.s006.docx]

**Table A in S1.**

**REPRODUCTIVE SPANS**

| **Genotype** | **Replicate**  **#** | **Mean days reproductive** | **% change** | **p-value** | **# of animals** |
| --- | --- | --- | --- | --- | --- |
| N2/*daf-2(e1370)* | 1 | 8.52/11.59 | +36.0 | <0.0001 | 96/121 |
|  | 2 | 9.18/11.75 | +28.0 | <0.0001 | 77/95 |
|  | 3 | 9.87/13.27 | +34.4 | <0.0001 | 81/97 |
| N2/*drp-1(tm1108)* | 1 | 8.52/6.05 | -40.8 | <0.0001 | 96/109 |
|  | 2 | 10.55/7.74 | -36.3 | <0.0001 | 59/75 |
|  | 3 | 7.46/3.37 | -121.4 | <0.0001 | 91/70 |
| N2/*fzo-1(tm1133)* | 1 | 7.46/5.64 | -32.3 | <0.0001 | 91/89 |
|  | 2 | 6.52/5.35 | -21.9 | <0.0001 | 81/79 |
|  | 3 | 9.87/ 8.75 | -12.8 | 0.0005 | 89/70 |
| *daf-2(e1370)*/  *daf-2(e1370);drp-1(tm1108)* | 1 | 11.59/9.35 | -24.0 | <0.0001 | 121/109 |
|  | 2 | 11.58/9.17 | -26.3 | <0.0001 | 109/100 |
| *daf-2(e1370)*/  *daf-2(e1370);fzo-1(tm1133)* | 1 | 13.27/12.41 | -6.9 | 0.7803 | 102/101 |
|  | 2 | 6.96/6.98 | +0.3 | 0.3391  n.s. | 97/99 |
| N2/*pink-1(tm1779)* | 1 | 7.86/ 7.67 | -2.5 | 0.7564  n.s. | 59/62 |
| N2  DMSO/UA 50µM | 1 | 9.1/10.4 | +14.29 | <0.0001 | 100/100 |
|  | 2 | 8.33/9.11 | +9.36 | 0.0002 | 97/100 |
|  | 3 | 9.1/10/39 | +14.8 | <0.0001 | 120/118 |
| *pink-1(tm1779)*  DMSO/UA 50µM | 1 | 9.18/9.39 | +2.29 | 0.4394  n.s. | 99/100 |
|  | 2 | 8.24/8.04 | -2.43 | 0.9404  n.s. | 100/99 |
| N2 Day 4 start  DMSO/UA 50µM | 1 | 9.1/9.4 | +3.3 | 0.0074 | 120/119 |
|  | 2 | 8.33/8.82 | +5.88 | 0.0062 | 97/98 |

**Table B in S1.**

**LATE MATINGS**

| **Genotype** | **replicate #** | **% reproductive** | **% difference** | **p-value** | **# of animals** |
| --- | --- | --- | --- | --- | --- |
| N2/*daf-2(e1370)* | 1 | 11.6/81.8 | +70.2 | <0.0001 | 69/74 |
|  | 2 | 34.3/68.2 | +33.9 | <0.0001 | 70/74 |
| *daf-2(e1370)*/  *daf-2(e1370);drp-1(tm1108)* | 1 | 56.3/16.9 | -36.4 | <0.0001 | 103/77 |
|  | 2 | 52.2/17.9 | -34.3 | <0.0001 | 90/67 |
| *daf-2(e1370)*/  *daf-2(e1370);fzo-1(tm1133)* | 1 | 56.3/60.8 | +4.5 | 0.5157  n.s. | 103/102 |
|  | 2 | 52.2/47.8 | -4.4 | 0.5510  n.s. | 90/93 |
| *daf-2(e1370)*/  *daf-2(e1370);pink-1(tm1779)* | 1 | 81.8/58.4 | -23.4 | 0.0025 | 74/77 |
|  | 2 | 68.2/41.4 | -26.8 | 0.0017 | 66/70 |
|  | 3 | 80.9/65.8 | -15.1 | 0.0093 | 115/120 |
| N2/*pink-1(tm1779)* | 1 | 47.3/50.6 | +3.3 | 0.6485  n.s. | 110/79 |
|  | 2 | 65.7/76.8 | +11.1 | 0.1161  n.s. | 99/99 |

**Table C in S1.**

**LIFESPANS**

| **Genotype** | **Replicate**  **#** | **Mean days**  **alive** | **% change** | **p-value** | **# of animals** |
| --- | --- | --- | --- | --- | --- |
| *daf-2(e1370)*/  *daf-2(e1370);drp-1(tm1108* | 1 | 39.71/41.49 | N/A | 0.4130  n.s. | 80/81 |
|  | 2 | 44.30/44.50 | N/A | 0.2320  n.s. | 78/71 |
| N2/*drp-1(tm1108)* | 1 | 15.50/15.10 | N/A | 0.4982  n.s. | 81/81 |
|  | 2 | 12.43/12.86 | N/A | 0.4291  n.s. | 62/46 |
| *daf-2(e1370)*/  *daf-2(e1370);fzo-1(tm1133)* | 1 | 39.46/44.91 | N/A | 0.1364  n.s. | 81/80 |
|  | 2 | 43.54/43.14 | N/A | 0.9177  n.s. | 84/83 |
| N2/*fzo-1(tm1133)* | 1 | 16.61/18.06 | N/A | 0.0889  n.s. | 80/82 |
|  | 2 | 11.84/12.68 | N/A | 0.6285  n.s. | 84/70 |
| *daf-2(e1370)*/  *daf-2(e1370);pink-1(tm1779)* | 1 | 43.43/41.54 | N/A | 0.8166  n.s. | 79/66 |
|  | 2 | 44.30/43.82 | N/A | 0.3965  n.s. | 78/74 |
| N2/*pink-1(tm1779)* | 1 | 15.82/14.80 | N/A | 0.6838  n.s. | 66/58 |
|  | 2 | 12.43/12.94 | N/A | 0.3861  n.s. | 62/69 |
| N2  DMSO/UA 50µM | 1 | 11.84/14.42 | +21.8 | <0.0001 | 80/80 |
|  | 2 | 13.4/12.03 | +11.39 | 0.0007 | 100/100 |
