# Supplementary material for: Oocyte mitophagy is critical for extended reproductive longevity
Source: PLoS Genet. 2022 Sep 20;18(9):e1010400. doi: 10.1371/journal.pgen.1010400 (PMC9524673; doi:10.1371/journal.pgen.1010400)
Supplement: Attachment — Submitted filename: reviewer response 080122ctm.docx [file pgen.1010400.s013.docx]

Thank you to the reviewers for a thorough reading and their important comments. Since our original submission, we have added the following experiments to address the reviewers’ questions:

1. Expression analysis of *fzo-1, drp-1*, and *pink-1* in *daf-2* mutants
2. Analysis of *sma-2* mitochondrial morphology
3. Progeny counts of N2, *fzo-1,* and *drp-1*
4. % Unhatched for N2, *drp-1*, and *fzo-1*
5. Mating capability analysis of *fzo-1*, *drp-1*, and double mutants with *daf-2*
6. Late-mated RS of *pink-1* mutants
7. Urolithin A treatment of d*af-*2 – Reproductive span analysis
8. Urolithin A treated*-drp-1* oocyte quality analysis
9. Urolithin A treatment of *daf-2;drp-1* and *daf-2;fzo-1* mutants - Reproductive span analysis

For clarity, we will begin our response with Reviewers 2 and 3 because their comments are brief, then address Reviewer 1’s comments.

Reviewer's Responses to Questions

**Reviewer #2**: With the first publication about daf-2 mutants living longer than wild type animals was a finding that was buried in the original paper reporting that these animals also reproduced for a longer period of time. Until the manuscript by Cota et al, the mechanism was unknown. The authors of this manuscript first find that the mitochondria found in the germline of daf-2 mutant animals are more punctate and the extended reproductive profile depends on active mitophagy. This is truly surprising because the mitochondria of the soma are elongated and trend towards fusion, rather than fission.

The authors do an excellent job of teasing apart the role for mitophagy in this process, using SKI-LODGE to assess mitophagy specifically in the germline, showing that pink1 is require for reproductive longevity, but not somatic longevity and finally, ectopic activation of mitophagy can increase the reproductive lifespan of wild type animals.

Thank you!

I am a huge fan of this paper and feel that no new experiments will strengthen this already strong paper.

Thank you! We appreciate this.

It is suggested that the authors qualify the use of Urolithin A in their studies as not solely targeting the germline since it is drug and hits all tissues.

Thank you, that is a good point and we have added this note to the text on page 12 lines 315-321.

**Reviewer #3**: In this very interesting manuscript, Cota et al delved into to the mechanisms of mitochondrial dynamics in regulating female reproductive longevity. Using daf-2 mutated C. elegans as a model, the authors found daf-2 mutant could extend reproductive span through shifting toward fission and activating mitophagy program to maintain oocyte quality. More importantly, they showed reproductive span could be extended by Urolithin A treatment.

The present paper is original, well performed, complete, and thoughtfully interpreted. The data provide a novel approach to promote oocyte health.

Thank you! We appreciate the reviewer’s positive comments.

It would strengthen the manuscript if the authors could address the following:

Major points:
To clarify the relationship of daf-2 mutant, fission/fusion, and mitophagy, a few experiments could be conducted.

1. To support daf-2 mutant could drive a shift towards fission and activate/strengthen the mitophagy program, it would be very feasible to compare the drp-1/fzo-1 gene expression ratio and pink-1 gene expression level in oocyte from daf-2 mutant and WT C. elegans.

Thank you for this question. Although *daf-2* is well known for its regulation through transcription, we hope that nothing in our text suggested that we expected these genes to be transcriptionally regulated, since they had not come to our attention based on our previous extensive analyses of *daf-2* and wild-type oocyte gene expression (Luo, et al. 2010; Templeman, et al 2018); our re-analysis here shows that none of these genes exceed any fold-change or significance values that would suggest major transcriptional regulation. Based on these oocyte expression data (Templeman, et al. 2018) from Day 8 hermaphrodites, we can see these genes do not appear to be regulated at the transcriptional level. However, there are many ways these proteins can be regulated post-translationally. We have now noted in the text (page 13 lines 370-372) that this regulation appears to be non-transcriptional.

2. In addition to abrogating the fission/fusion, the approach that the authors used, mimicking punctate/elongated morphology would further support that fission-mediated punctate is required for mitophagy. For example, knockdown of drp-1 or overexpression of fzo-1 in daf-2 mutant C. elegans would elongate mitochondria and thus confer shorter reproductive span?

That was exactly our expectation as well, and is what we showed in Figure 3; either a decrease in *drp-1* or an increase in FZO-1 would be expected to force *daf-2* oocytes to shift to elongated mitochondria, which we showed in Figure 3A, and this shift to elongated mitochondria also decreases oocyte quality (Figure 3G, H) and shortens reproductive span (Figure 3D).

We have now added the parallel data for *daf-2;fzo-1* (Figures 3E, F, I) to contrast with *daf-2;drp-1*, underscoring the point that the punctate mitochondria of *daf-2* oocytes maintains oocyte quality.

Minor concerns:
1. Please add the missing scale bar in Figure 1C, Figure 3G, and Figure 3H.

Thank you for noting this omission; we have now fixed it.

**Reviewer #1**: In the Cota et al manuscript, the authors used the daf-2 mutant in C. elegans as a model to study the role of mitochondrial fragmentation and increased mitophagy in preserving oocyte quality towards the end of the reproductive period. Overall, the paper reported a number of interesting findings, but additional information is required to solidify the conclusions the authors put forth.

Specific comments:
1) It was previously reported that daf-2 mutants have extended reproductive span, but they also have reduced brood size. In this study, the authors performed the experiments using mated daf-2 mutants. Please include quantification of oocyte quality using unmated daf-2 as control (as in Fig 1C).

**Reproductive assays in *unmated* animals cannot produce not a true measurement of oocyte quality maintenance**, and cannot act as a control for mated assays, because they are subject to several confounding factors unrelated to oocyte quality. Hermaphrodites produce a limited number of sperm, which dictates the number of progeny and the reproductive span in a selfed (unmated) assay; in fact, because sperm rather than oocytes are limiting in self-mated assays, unmated assays only occasionally reflect oocyte quality. Because selfed (unmated) reproductive spans can be limited by sperm number, by sperm quality, or by oocyte quality, what *exactly* is being measured in an unmated RS assay is always unclear.

Furthermore, in an unmated assay of a mutant, both sperm and oocytes are mutant; therefore, one cannot distinguish between the genetic effects of the mutation on sperm or oocytes in an unmated reproductive span assay. **Mated assays that use young wild-type males are in fact the control for unmated reproductive assays**, as they isolate oocyte function from sperm function, which are convoluted in unmated assays. By mating mutants of interest with young wild-type sperm we remove the *variable* of sperm to 1) get a true measurement of reproductive decline based on oocytes and 2) remove the confounding factor of possible impairment of mutant sperm. Therefore, only mated assays can truly assess oocyte quality.

Although *daf-2’s* extended unmated reproductive span had already been shown (Huang, et al. 2004), Hughes et al. 2007 reported *daf-2* has extended reproductive longevity when hermaphrodites are not limited by sperm number. This work was expanded on in our lab when we showed reproductive decline is dictated by oocyte quality maintenance (Luo et al. 2009 and Luo, et al. 2010), which we further assessed in *daf-2* mutants (Templeman, et al. 2018).

Here we have used the appropriate controls (mutant against wild-type, double mutants vs single mutants) in mated assays throughout the work to determine the effect of specific genes specifically on oocyte quality maintenance with age.

2) Are fragmented mitochondria (in oocytes) observed in other mutants with extended reproductive span, e.g., TGF beta mutants?

Although the analysis of mutants in a separate pathway is beyond the scope of the current manuscript (it is the focus of a different study), we have found that *sma-2* mutants look like wild-type oocytes both when young and old (both display networked mitochondria), unlike *daf-2* mutants (whose mitochondria are punctate in both young and old oocytes), suggesting non-mitochondrial morphology mechanisms extend *sma-2* reproductive span, as we previously reported (Luo et al. Cell 2010). Although we previously found that there are transcriptional similarities between *sma-2* and *daf-2* oocytes (Luo, et al. 2010), we have also found insulin-signaling-specific mechanisms (Templeman, et al. 2018), and these shifts in mitochondrial morphology appear to be an IIS-specific mechanism to maintain oocyte quality.

3) Fig 1B showed late mating capability. It’d be important for the authors to demonstrate the non-reproductive worms were mated successfully.

In full reproductive spans we confirm the mating success of each individual hermaphrodite based on the presence of male progeny. The graph below is based on the data from those experiments. L4 hermaphrodites were added on the plate in a 2:1 ratio with *fog-2* males (late-mating assays are in a 3:1 male:herm ratio). After approx. 24 hrs with males, hermaphrodites are singled and lay progeny for 24 hours. Two days later progeny is scored and considered “successfully mated” if approx. 1/2 progeny is male. **Each mutant used in this paper has similar mating ability to their appropriate controls (wild type or *daf-2(e1370),* graph below).** Each late mating assay has been repeated 2-3 times with similar results with large n’s (see supplemental table 2). We have added these data to the paper (Figure S4B).

We use late mating to test the oocyte quality and mating capability of animals after their own sperm have been used up, since only aged oocytes will be present at that time. Not only have we found that this assay has always reflected the same effect on reproductive span as does the full reproductive span (Templeman, et al. 2018), late mating has the advantage that it avoids matricide problems that are caused by early mating with males. In many cases, because matricide rates are high in specific mutants (due to egg laying and/or lifespan uncoupling problems that are not related to oocyte quality), late mating is the only assay that can be done. Matricide often happens when the reproductive span and oocyte quality maintenance of a mutant exceed the concomitant increase in somatic health. For example, *sma-2* worms have normal lifespans but greatly extended reproductive spans, and almost all of the animals bag (Luo, et al. 2010). This is the case for N2 vs. *daf-2* (Fig 1A compare to Fig 1B), *daf-2* vs. *daf-2;drp-1* (Figure 3D compare to Fig 3F), and *daf-2* vs. *daf-2;fzo-1* (Fig 3E compare to Fig 3F). Late mating, therefore, allows us to accurately measure reproductive span as a reflection of the quality of aged oocytes that avoids matricide.

4) In Figure 2, the ovulation rate and total brood size of drp-1 and fzo-1 mutants need to be shown. These are important controls and would help interpret the observed reduced reproductive span phenotype. (It was previously reported that drp-1 depletion reduces brood size and increases lethality.)

Thank you for the suggestion. It is true that Kanazawa, et al. (2008) showed reduced brood sizes of unmated *fzo-1(RNAi)* and *drp-1(RNAi)* worms when compared to control N2; however, it is unclear from their unmated assays whether the reduced progeny is due to sperm defects (function or sperm number) or oocyte quality. To determine whether this is an oocyte quality phenotype, we have mated *drp-1* and *fzo-1* mutants with wild-type males, and we find that the number of progeny is significantly reduced (2-way ANOVA) in the *drp-1* (n=13) and *fzo-1* (n=12) mutants compared to the N2 (n=12) control on Day 3 and Day 4 of adulthood after mating (L4-Day 1) and after confirming they are mated (Day 1-Day 2). Therefore, we can conclude from our mated assays that the reduced progeny number in the fission and fusion mutants during mid-reproduction are in fact due to oocyte quality issues. We have added these data (Figure S2C).

5) In Figure 3D, E and FM, it’d be important to include the reproductive span and late mating capability of drp-1 and fzo-1 single mutants as controls.

We have now included these data (Figure S3C and S3D); our original conclusions are unchanged. (Late mating was not necessary in these cases, as we were able to complete full reproductive spans without high bagging rates.) To avoid confusing readers, we will highlight the proper genetic comparison (*daf-2* vs *daf-2; drp-1* and *daf-2* vs *daf-2;fzo-1*) in the main figures, since the other data (N2, *drp-1*, and *fzo-1*) are consistent and redundant with data shown in Figure 2D and E, and are not the main point of the experiment here.

6) Figure 2 showed that day-6 drp-1 and fzo-1 mutants develop severe oocyte aberration. These phenotypes should also be assessed for the daf-2 and daf-2; fzo-1 mutants in Figure 3 G, H and I.

We did show these data (shown here again on the right) in the original Supplemental Figure 4; as we stated, there is no defect in oocyte quality with age when comparing *daf-2;fzo-1* vs. *daf-2* control. To avoid the readers missing this point, we have moved these data from the Supplement to the main figures (now Figure 3I).

7) For Figure 3J, the data for unhatched oocytes in daf-2;fzo-1 and single mutants, drp-1 and fzo-1, need to be included as appropriate controls.

We have now included these data (Figure 2F). Both *fzo-1* and *drp-1* have significantly more unhatched oocytes, correlating with their shortened reproductive spans (Figures 2D and 2E) and suggesting that both fission and fusion are required for wild-type oocyte quality, as we had previously concluded from our reproductive span and oocyte quality assays data.

Note however that *daf-2;fzo-1* mutants do not exhibit signs of reproductive decline (no difference from *daf-2* in mated RS (Figure 3E), same late mating capability (Figure 3F), and no difference in oocyte quality with age (Figure S4), underscoring the importance specifically of the punctate morphology of mitochondria in *daf-2* oocytes.

8) In Figure 4, reproductive span data for daf-2 and daf-2;pink-1 should be included in parallel to late mating capability data in figure 4A as these two experiments have been shown together in most of the figures to measure reproductive span and oocyte quality.

We directly measured oocyte quality, as we showed in Figure 4b and 4c; *daf-2;pink-1* mutants have more uterine masses, as we also observed with *daf-2;drp-1*, suggesting that loss of *pink-1* and *drp-1* have similar effects on *daf-2* oocyte quality. We will make this point clearer to readers (page 10, lines 266-269).

As we explained in point 3 above, late mating assays are analogous to full reproductive spans (compare Fig 1A to 1B, compare Fig 3D and E to 3F), but avoid the high censoring rate due to matricide (“bagging”) that is caused by extended reproduction (Templeman, et al. 2020). We have tried replicates of the full mated reproductive span of *daf-2* vs. *daf-2;pink-1*, but the vast majority of animals “bag,” therefore we used late mating assays to assess mating capability, and oocyte quality assays to assess oocyte quality. We have made this point in the text (page 10, lines 264-266)

9) In Figure 4, pink-1 single mutant data for oocyte quality check (uterine mass quantification), reproductive span and late mating capability need to be included as appropriate controls.

We were interested in whether PINK-1 plays a role in *daf-2*’s extended reproductive longevity, therefore loss of PINK-1 in the *daf-2;pink-1* double mutant should be compared to *daf-2* as the control. We see no major defect in pink-1 in an otherwise wild-type background, suggesting that its role becomes important as oocyte usage is extended in daf-2 mutants. As mentioned in point 8, the *daf-2;pink-1* double mutant exhibits high matricide rates in mated reproductive spans. However, when we investigated *pink-1* alone, compared to the N2 control, to ask whether PINK-1 plays a role in the reproductive span of wild type, we found no change in reproductive span (Fig 4D). This was confirmed with late-mating results (data below and now included in Figure S4C, N2 vs. *pink-1*). When compared to the N2 control, the reproductive span and late mating results for *pink-1* mutants suggest there is no defect in oocyte quality decline relative to wild-type, suggesting that, like its punctate mitochondrial morphology, *pink-1*-mediated mitophagy is uniquely required for *daf-2’s* extended reproductive span and oocyte quality.

(10) The extended reproductive span of daf-2 worms depends on punctate mitochondrial morphology and mitophagy in the oocytes. In figure 4, it is shown that blocking mitophagy by depleting PINK-1 causes severe oocyte anomalies in day-8 daf-2 worms. These results suggest that pink-1 might be overexpressed or mitophagy is increased in the oocytes of daf-2 mutants. It would be important to include pink-1 expression data in daf-2 oocytes.

As we showed in the response to Reviewer 3, we do not have any data supporting (nor did we suggest) the idea that *pink-1* is regulated transcriptionally by *daf-2*; we apologize if that was implied, and we have now noted in the text (page 13 lines 370-372) that this regulation appears to be non-transcriptional.

(11) In figure 5, the effect of Urolithin (a mitophagy promoter metabolite) on reproductive span of daf-2 mutant should be included as a control.

The point of the UA experiments was to understand whether mimicking the punctate-and-mitophagy strategy that *daf-2* uses could improve *wild-type worms’* reproduction, as it does. DMSO, the diluent for UA, is the proper control for all UA treatments (as UA is dissolved in DMSO) and is therefore the control we used in all UA experiments.)

UA treatment did not change the reproductive span of *daf-2* mutants. These data are now shown in Figure S5B.

(12) In figure 5, it is shown that UA supplementation reduces the severity of uterine mass in fzo-1 mutants. Interestingly, drp-1 mutants also show severe uterine mass in day-6 worms (figure 2G). The authors should check the effect of UA supplementation in drp-1 mutants to specifically test whether the increased mitophagy upon excessive fragmentation (achieved by fzo-1 depletion) is the underlying mechanism for better oocyte quality in daf-2 mutants. If UA supplementation also reduces the severity of uterine mass in drp-1 mutants, then it would suggest a more general role of UA in regulating oocyte quality irrespective of the mitochondrial morphology in the oocytes.

Thank you, this was a fantastic suggestion. Our new data suggest that UA does not improve the oocyte quality of *drp-1* mutants, arguing against a general role of UA and instead supporting our original conclusion that *daf-2* uniquely shifts toward punctate mitochondria in order to use mitophagy to improve oocyte quality (whereas *fzo-1* mutants, which are already punctate, can take advantage of UA-induced mitophagy, Figure 5G). We have now added these data to Figure 5I.

(13) In Figure 5, the oocyte quality data upon UA supplementation in daf-2, daf-2;drp-1 and daf-2;fzo-1 on day 6 worms should be included as controls.

We observe that there is minimal improvement to the RS of *daf-2;drp-1*, while *daf-2;fzo-1* appears to be maximally extended already; the UA effect in *daf-2;drp-1* is significant but nowhere near the extension we see in *daf-2* mutants, which makes sense if only a few mitochondria are in the punctate form that is necessary for UA-induced mitophagy.

Minor:
In line 261, Figure 6F-G should be corrected to 5F-G.

Thank you for noticing these errors, we have now corrected them.
